# High-Grade Tumor Budding Stratifies Early-Stage Cervical Cancer with Recurrence Risk

**DOI:** 10.1371/journal.pone.0166311

**Published:** 2016-11-18

**Authors:** Bangxing Huang, Jing Cai, Xia Xu, Shuang Guo, Zehua Wang

**Affiliations:** 1 Department of Gynecology and Obstetrics, Union Hospital, Tongji Medical College, Huazhong University of Science and Technology, Wuhan, Hubei, China; 2 Department of Pathology, Union Hospital, Tongji Medical College, Huazhong University of Science and Technology, Wuhan, Hubei, China; Taipei Medical University, TAIWAN

## Abstract

**Objectives:**

This study investigated prognostic significance of tumor budding in early-stage cervical cancer (ESCC) following radical surgery and its contribution to improve the stratification of patients with recurrence risk.

**Methods:**

The archival medical records and H&E-stained slides of 643 patients with IA2-IIA stage cervical cancer who underwent radical surgery were retrospectively reviewed. Clinicopathological parameters were noted, and tumor buds were counted using immunohistochemistry for each case. The prognostic significance of tumor budding was analyzed. Prediction models that comprised tumor budding were established, and the performance was compared between the novel models and classic criteria via log-rank test and receiver operating characteristic analysis.

**Results:**

Tumors with high-grade tumor budding (HTB) exhibited a substantially increased risk of recurrence (hazard ratio = 4.287, *P* < 0.001). Nine predictive models for recurrence were established, in which HTB was combined with recognized risk factors. The model using of at least two risk factors of HTB, tumor size ≥ 4 cm, deep stromal invasion of outer 1/3, and lymphovascular space invasion to stratify patients with an intermediate risk was most predictive of recurrence compared with the classic criteria.

**Conclusions:**

Tumor budding is an independent, unfavorable, prognostic factor for ESCC patients following radical surgery and holds promise for improved recurrence risk stratification.

## Introduction

In low-income and middle-income countries, cervical cancer is a leading cause of cancer-related deaths in women [1]. In China, the estimated cervical cancer incidence and mortality rates per 100,000 population new cases and deaths in 2011 were 13.40 and 3.56, respectively [2]. More than 80% of cervical cancers were diagnosed at a local or regional stage, and the clinical outcomes varied [3]. Primary radical hysterectomy with pelvic lymphadenectomy is the treatment of choice for patients with early-stage cervical cancer (ESCC). Following radical surgery, doctors must decide whether to administer adjuvant treatment for each individual patient. Postoperative adjuvant radiation or chemoradiation may significantly reduce the risk of disease progression at 5 years; however, it substantially increases complications and impacts quality of life in patients with ESCC [4–6]. It is therefore important to stratify these patients regarding their risk of recurrence to avoid overtreatment and minimize treatment-related morbidity in patients with a low risk.

A series of pathological factors, such as pelvic lymph node metastasis (PLNM), positive surgical margin, parametrium involvement, deep stromal invasion (DSI, outer half or outer third of cervical stroma), lymphovascular space invasion (LVSI), large tumor size (e.g., ≥ 2 cm or ≥ 4 cm in diameter), tumor grade, and non-squamous histological subtype, have been associated with the risk of recurrence following radical surgery [6]. Of these variables, PLNM, parametrial involvement, and positive surgical margin have been identified as high-risk factors, and the remaining variables have been identified as intermediate-risk factors according to the magnitude of their impacts on the recurrence rate and the disease-free interval [6–8]. Adjuvant radiation or chemoradiation is indicated for patients with any of the three high-risk factors. However, the stratification criteria of the intermediate risk group have been debated.

In the “classic criteria” [9, 10], DSI, LVSI and tumor size ≥ 2 cm are used to stratify ESCC with an intermediate risk to envelop a recurrence: patients with two or more of these factors are candidates for adjuvant treatment; these women have a 31% probability of cancer recurrence at 3 years. Moreover, 25% of stage IB cervical cancer patients with negative lymph nodes meet the classic criteria, which indicate a limited specificity of the Classic criteria [9, 11]. Sedlis criteria (also referred to as GOG criteria) are also based on these three intermediate-risk factors using various combinations: a) any tumor size with LVSI and stromal invasion of deep 1/3; b) LVSI, stromal invasion of middle 1/3, and tumor size ≥ 2 cm; c) LVSI, stromal invasion of superficial 1/3, and tumor size ≥ 5 cm; or d) stromal invasion of middle or deep 1/3 and tumor size ≥ 4 cm. The recurrence rate in patients with stage IB cervical cancer who meet the Sedlis criteria was 28% without additional treatment, and an adjuvant pelvic radiotherapy achieved a reduction of 47% in the risk of recurrence in these patients [4]. Recently, a “four-factor model” has been identified, in which the presence of two of four intermediate-risk factors (tumor size ≥ 3 cm, DSI of the outer third of the cervix, LVSI, and adenocarcinoma or adenosquamous carcinoma histology) may be used to predict recurrence in ESCC patients with improved performance compared with both the “classic” and Sedlis criteria [10]. These findings indicate that additional risk factors other than the classic risk factors may be helpful to stratify patients with a recurrence risk.

Tumor budding was initially termed sprouting by Imai in the 1950s. Morphologically, tumor budding is defined as the presence of single tumor cells or isolated small clusters of tumor cells (up to 5 tumor cells) scattered in the stroma at the invasive front; it represents a type of local diffusely infiltrative growth and occurs when tumor cells detach from the invasive tumor margin and migrate into the surrounding stroma. Tumor budding has frequently been observed in several types of solid tumors, including colorectal cancer [12], esophageal carcinoma [13], pancreatic carcinoma [14, 15], breast cancer [16, 17], lung carcinoma [18, 19], and endometrial carcinoma [20]; tumor budding has been recognized as an aggressive indicator and an adverse prognostic factor [12–20]. Moreover, as a result of its significant prognostic value, tumor budding is recommended by the International Union against Cancer for inclusion in the pathological report of colorectal cancer as an additional prognostic factor [21]. In cervical cancer, the spray-like invasion pattern has been considered as an adverse prognostic factor [22]. However, tumor budding is a pathological term distinguished from infiltrative patterns (infiltrative, finger-like, spray-like, etc.) [23, 24], and the occurrence and clinical relevance of tumor budding in cervical cancer remains unexplored.

In the present study, we aimed to investigate the association between tumor budding and recurrence in patients with ESCC following radical surgery and to assess the contribution of tumor budding in improving the accuracy regarding the prediction of recurrence.

## Materials and Methods

### Case selection and clinicopathological review

We reviewed the archival medical records and H&E-stained slides of cervical cancer patients at Union Hospital, Tongji Medical College, Huazhong University of Science and Technology. ESCC (FIGO stage IA2-IIA) patients who had undergone primary radical hysterectomy with adequate pelvic lymphadenectomy (the number of nodes removed ≥ 10 or pathologically confirmed positive nodes) between January 2008 and December 2014 were enrolled in the present study. Patients with a history of preoperative chemotherapy or radiotherapy or synchronous malignancies were excluded. In addition, cases that lacked clinicopathological or follow-up data were excluded as not assessable. The following clinicopathological parameters were noted: age at diagnosis, FIGO stage, tumor size, histological subtype, histological grade, depth of stromal invasion, parametrial involvement, surgical margin status, lymph node status, and LVSI. Histopathologic types and grades were categorized according to the WHO classification of tumors of female reproductive organs (4th Edition, 2014). This retrospective study was approved by the local ethics committees of Huazhong University of Science and Technology, and it was conducted in accordance with the guidelines of Declaration of Helsinki from January 2015. The patient records were retrospectively reviewed. The data regarding tumor characteristics, management and treatment outcomes were collected and anonymized data were used in subsequent statistical analysis. Each subject provided written informed consent before enrollment.

### Assessment of tumor budding using immunohistochemistry

To optimally visualize tumor buds and to distinguish budding cells from stromal or inflammatory cells, our assessment is based on immunohistochemical evaluation. All available H&E-stained slides were scanned to select one tissue block showing maximal budding for each case. The corresponding tissue blocks were subsequently used to perform immunohistochemistry with anti-human pan-cytokeratin antibodies to highlight buds. The entire cytokeratin-stained slides were first scanned at low power magnification to identify the areas with the most intense buds. Hot-spots of tumor budding were evaluated and the number of buds was subsequently counted in a field that measured 0.95 mm^2^ using a ×20 objective lens (Olympus, BX-51). For each case, buds were counted in ten high power fields (HPF), and the maximum bud count per HPF was used for the budding grade evaluation. Two experienced gynecologic pathologists scored tumor budding that blinded to the clinicopathological data and outcomes. There were 57 inter-observer discrepancies in the quantitative assessment of tumor budding; the discrepancies were resolved by consensus using a multihead microscope.

For pan-cytokeratin immunohistochemistry, whole 4-*μ*m tissue sections were de-waxed, rehydrated in an ethanol gradient, and rinsed with phosphate-buffered saline (PBS). Following pressure cooker-mediated antigen retrieval in citrate buffer (pH 6.0) for 1 min and 30 s, the endogenous peroxidase activity and non-specific binding were blocked using 1% H_2_O_2_ and 1% bovine serum albumin (BSA) for 20 min at room temperature. The slides were subsequently incubated with mouse anti-human pan-cytokeratin antibody (clone AE1/AE3; dilution 1:100; DakoCytomation, Denmark) overnight at 4°C. Immunodetections were performed using the DaKo EnVision Kit (DakoCytomation, Denmark), and Diaminobenzidine (DakoCytomation, Denmark) was used as the chromogen. Negative controls (primary antibodies replaced by PBS buffer) were conducted to exclude the possibility of non-specific staining.

### Patient management

The preoperative evaluation procedure, the radical hysterectomy technique, and the follow-up strategy have been described in our previous study [25]. Briefly, all patients received laparoscopic radical hysterectomy and systematic bilateral pelvic lymphadenectomy. Adjuvant radiation or chemoradiation was recommended for the patients with ≥ 1 high-risk factors (i.e., lymph node metastasis, parametrial involvement, and positive surgical margins) and the patients with ≥ 2 intermediate-risk factors (i.e., LVSI, DSI, and tumor size ≥ 2 cm). Following treatment completion, the patients were subsequently followed every 3 months for the first 2 years, every 6 months in the third year, and annually thereafter until the end of the study. The follow-up methods included an interval history, gynecological examination, vaginal stump brushing cytology, chest X-ray and pelvic and abdominal ultrasonography. Imaging (computed tomography, positron emission tomography and magnetic resonance imaging) and colposcopy with directed biopsy were performed if indicated based on symptoms or examination findings suspicious for recurrence. The enrolled patients were followed until December 31, 2015.

### Statistical analysis

All statistical analyses were performed using SPSS software (version 19.0, Chicago, IL, USA). All analyses were 2-sided, and *P* values less than 0.05 were considered statistically significant.

Data were descriptively summarized using frequencies and percentages for the categorical variables and medians and ranges for the continuous variables. Associations between the number of buds (as a continuous variable) and the categorical clinicopathological parameters were examined using the Wilcoxon signed-rank test (the Mann–Whitney U test or the Kruskal–Wallis H test, as appropriate). A receiver operating characteristic (ROC) curve analysis was performed to assess the performance of bud counts to discriminate recurrences from non-recurrences and to determine a valid cut-off value to divide patients into a high-grade tumor budding (HTB) group and a low-grade tumor budding (LTB) group. In addition, ROC analyses were used to compare the performance of HTB with the recognized risk factors for recurrence in ESCC following radical surgery.

Univariate and multivariate Cox proportional hazard models were used to determine the association between potential risk factors and recurrence, as well as death from disease. The potential risk factors included in the univariate Cox analysis were age at diagnosis, histological subtype, histological grade, FIGO stage, tumor size, DSI, parametrium involvement, surgical margin status, PLNM, LVSI, and HTB; the factors significantly associated with outcomes in the univariate analysis were considered for inclusion in the multivariate stepwise logistic regression analysis. Disease-free survival was defined as the time from primary surgery to recurrence diagnosis; overall survival was defined as the time from primary surgery to death from disease. Data from the patients who did not experience the event were censored at the date of last follow-up. Hazard ratios (HR) and their 95% confidence intervals (CI) for the dichotomous categorical variables were used to assess the independent contributions of significant factors.

Kaplan-Meier survival analyses were used to estimate the disease-free survival function; log-rank tests were used to identify the statistical significance of the differences between the subgroups. We established nine models that included HTB and the classic risk factors to stratify the ESCC patients with recurrence risk; a log-rank test and ROC analysis were used to compare their performance with the classic criteria.

## Results

### Tumor budding in ESCC

**Fig 1:**
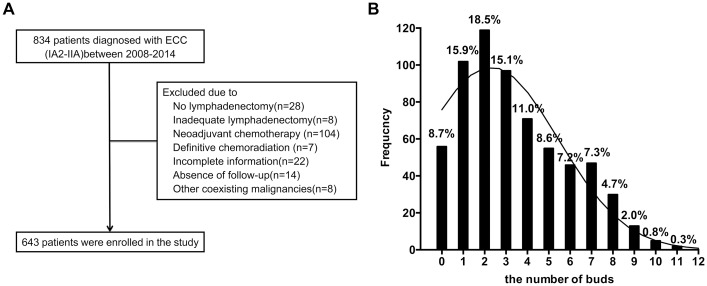
Participant flow and the frequency of tumor budding. A, patient selection flow chart. B, patient distribution by the intensity of tumor buds, counted in a microscopic field of 0.95 mm^2^.

Eight hundred thirty-four patients were diagnosed with FIGO stage IA2-IIA cervical cancer between January 2008 and December 2014 at our hospital. Six hundred forty-three patients satisfied the eligibility criteria and were enrolled in the present study (Fig 1A). The clinicopathological findings of these patients (S1 File) are summarized in Table 1. Following surgery, according to our institute criteria, adjuvant therapy was indicated for 246 patients (38.26%) with one high-risk factor (N = 106) or with two intermediate-risk factors (N = 140). However, only 137 (21.30%) patients underwent adjuvant therapy; nearly half of the patients with recurrence risk refused postoperative treatment for economic reasons or angst regarding treatment-related adverse effects. With a median follow-up time of 37 months (range, 11–97 months), 109 patients (16.95%) experienced recurrence, and 54 patients (8.40%) died. The estimated disease-free survival and overall survival rates were 85% and 91%, respectively, at 3 years, compared with 77% and 87%, respectively, at 5 years.

**Table 1:** Correlations between tumor budding and clinicopathological characteristics (N = 643).

Variables	N	The number of buds		*P* value
		Median	Q1-Q3	
Age (year)				0.775^a^
< 45	342	3	2–5	
≥45	301	3	1–5	
Histological type				0.088^b^
Squamous cell cancer	533	3	2–5	
Adenocarcinoma	82	2	1–4	
Others	28	3	2–7	
Histological grade				0.008^b^
Well differentiated	126	2	1–4	
Moderately differentiated	320	3	2–5	
Poorly differentiated	197	3	2–5	
FIGO stage				0.004^b^
IA	60	2	1–3	
IB1	487	3	2–5	
IB2	19	3	2–5	
IIA	77	3	2–6	
Tumor size (cm)				0.002^b^
< 2	267	2	1–4	
≥ 2 and < 4	327	3	2–6	
≥ 4	49	3	2–5	
Depth of stromal invasion				0.022^a^
Inner 2/3	477	3	1–5	
Outer 1/3	166	3	2–6	
LVSI				<0.001^a^
Negative	522	3	1–4	
Positive	121	5	3–7	
PLNM				<0.001^a^
Negative	550	3	1–4	
Positive	93	5	3–7	
Parametrium involvement				0.748^a^
Negative	631	3	2–5	
Positive	12	2.5	1–5	

^a^, Mann-Whitney U test;

^b^, Kruskal-Wallis H test.

LVSI = lymphovascular space invasion; PLNM = pelvic lymph node metastasis; Q = quartile.

**Fig 2:**
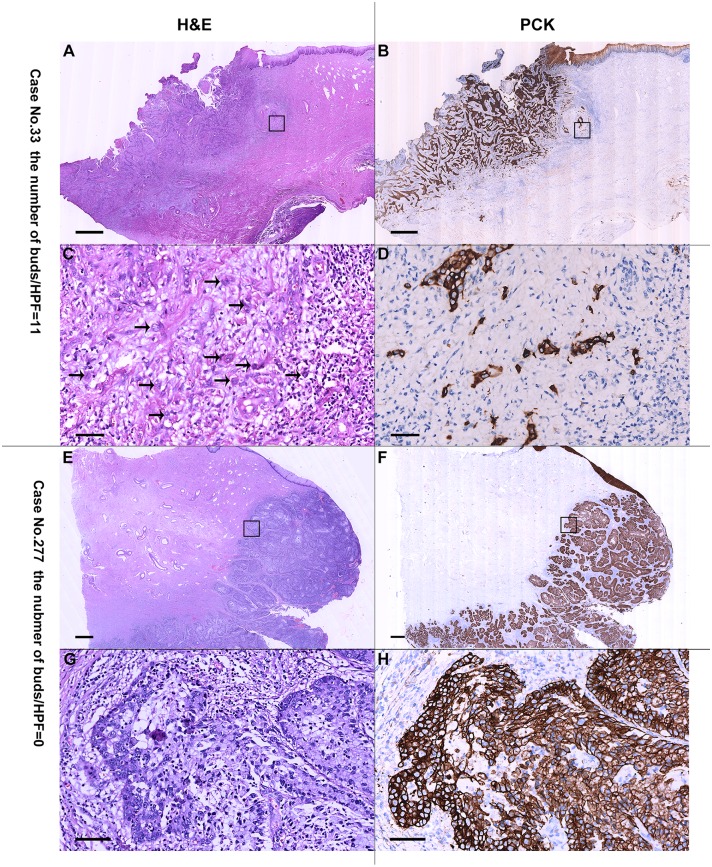
Representative areas of tumor buds in the outer edge of ESCC stained with H&E (A, C, E, and G) and pan-cytokeratin antibody (B, D, F, and H). A-D, a cervical cancer with intensive tumor buds (arrow); E-H, a cervical cancer with no bud. Scale bar is 1 mm in A, B, E and F and 50 μm in C, D, G and H.

In these patients, the number of tumor buds ranged from 0 to 11 with a median of 3 (Fig 1B). Fig 2 illustrates representative photomicrographs of a cervical cancer with intensive tumor budding and a cervical cancer with an absence of budding. The bud count was significantly correlated with worse tumor differentiation (*P* = 0.008), higher FIGO stage (*P* = 0.004), larger primary tumor (*P* = 0.002), DSI (*P* = 0.022), and the presence of PLNM and LVSI (both *P* < 0.001; Table 1).

### Tumor budding is an independent predictor for recurrence

**Fig 3:**
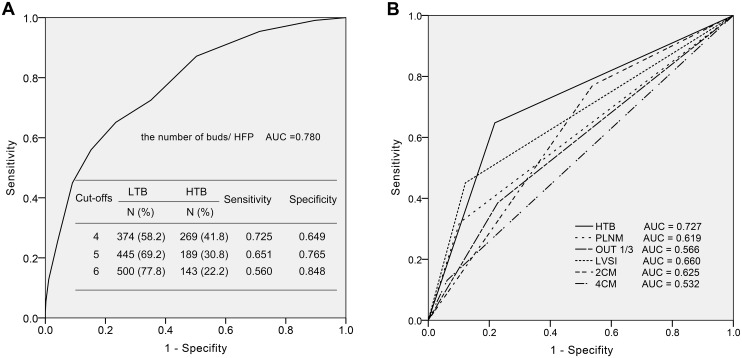
Receive operating characteristic (ROC) curves for the accuracy of disease recurrence. A, ROC curve aiding the selection of the cut-off value for low-grade budding (LTB) and high-grade budding (HTB). A value of 5 buds was selected because of its high sensitivity and specificity. B, ROC curves using various clinicopathological risk factors. HTB exhibited higher areas under the curve (AUC = 0.727) than the other classic clinicopathological risk factors. Abbreviations: OUT 1/3, stromal invasion of the outer 1/3; 2CM, tumor size ≥ 2 cm; 4CM, tumor size ≥ 4 cm.

The ROC curve analysis indicated that the number of buds offered an area under the curve (AUC) of 0.780 to distinguish the patients with recurrences from the patients with non-recurrences; these findings suggest that tumor budding is highly predictive of recurrence in patients with ESCC (Fig 3A). Five was selected as the cut-off value to divide the patients because 5 buds exhibited the highest accuracy with a sensitivity of 65.1% and a specificity of 76.5%. The patients were subsequently dichotomized into LTB group (0–4 buds, N = 445, 69.2%) and HTB group (5 or more buds, N = 198, 30.8%). Compared with the classic risk factors, i.e., large primary tumor (≥ 2 cm or ≥ 4 cm in diameter), DSI of the outer 1/3, LVSI and PLMN, HTB had the maximum AUC (0.727); tumor size ≥ 2 cm exhibited the highest sensitivity and the lowest specificity; tumor size ≥ 4 cm, PLNM, and LVSI exhibited a higher specificity but lower sensitivity (Fig 3B). Parametrium involvement and positive surgical margin were not included in the ROC analysis because of the limited case numbers (12 and 15 cervical cancers, respectively).

The univariate Cox regression analysis indicated that HTB, higher FIGO stage, larger primary tumor, DSI, PLNM, LVSI, parametrium involvement, adjuvant therapy and positive surgical margin were risk factors for both disease-free survival and overall survival (Table 2). These risk factors were subsequently included in the multivariate Cox regression analysis, and the results indicated that HTB (HR = 4.287, *P* < 0.001), as well as LVSI (HR = 2.210, *P* = 0.001), PLMN (HR = 1.936, *P* = 0.012), positive surgical margin (HR = 2.530, *P* = 0.037), and adjuvant therapy (HR = 0.576, *P* = 0.030) were independent prognostic factors for recurrence (Table 2). Moreover, HTB, similar to PLNM (HR = 3.713, *P* < 0.001) and LVSI (HR = 2.426, *P* = 0.013), was an independent risk factor for OS with a HR of 2.868 (*P* = 0.014; Table 2).

**Table 2:** Univariate and multivariate survival analysis for disease-free survival (DFS) and overall survival (OS).

Variable	Univariate DFS analysis		Multivariate DFS analysis		Univariate OS analysis		Multivariate OS analysis	
	HR (95%CI)	*P* value	HR (95%CI)	*P* value	HR (95%CI)	*P* value	HR (95%CI)	*P* value
Age (y) < 45 vs. ≥ 45	0.790 (0.550–1.137)	0.205	NA		0.639 (0.365–1.118)	0.116	NA	
SCC vs. ADE	0.856 (0.515–1.451)	0.582	NA		0.367 (0.132–1.016)	0.054	NA	
Poorly vs. well-moderately differentiated	1.005 (0.688–1.467)	0.980	NA		1.673 (0.904–3.095)	0.101	NA	
Tumor size (cm) ≥ 2 vs. < 2	2.797 (1.765–4.431)	0.000	1.701 (0.992–2.917)	0.053	2.441 (1.285–4.636)	0.006	1.101 (0.493–2.459)	0.841
Tumor size (cm) ≥ 4 vs. < 4	2.269 (1.293–3.983)	0.004	1.483 (0.775–2.838)	0.234	3.052 (1.491–6.248)	0.002	1.843 (0.762–4.457)	0.175
FIGO stage IB2-IIA vs. IA2-IB1	2.396 (1.548–3.710)	0.000	1.185 (0.708–1.984)	0.517	2.598 (1.410–4.785)	0.002	1.150 (0.555–2.383)	0.707
DSI out1/3 vs. inner 2/3	1.707 (1.156–2.520)	0.007	1.170 (0.716–1.911)	0.530	1.951 (1.133–3.358)	0.016	1.840 (0.887–3.816)	0.101
DSI out1/2 vs. inner 1/2	2.278 (1.543–3.363)	0.000	1.046 (0.612–1.788)	0.869	2.174 (1.251–3.778)	0.006	1.328 (0.597–2.952)	0.487
HTB vs. LTB	5.975 (3.989–8.951)	0.000	4.287 (2.757–6.667)	0.000	4.706 (2.687–8.239)	0.000	2.868 (1.509–5.451)	0.001
LVSI positive vs. negative	4.699 (3.214–6.870)	0.000	2.210 (1.366–3.575)	0.001	6.942 (4.053–11.890)	0.000	2.426 (1.197–4.918)	0.014
PLNM positive vs. negative	4.401 (2.939–6.590)	0.000	1.936 (1.155–3.246)	0.012	8.907 (5.176–15.327)	0.000	3.713 (1.812–7.608)	0.000
PI positive vs. negative	3.488 (1.421–8.565)	0.006	1.250 (0.479–3.259)	0.649	5.422 (1.956–15.030)	0.001	1.500 (0.495–4.541)	0.473
Adjuvant therapy yes vs. no	0.448 (0.301–0.666)	0.000	0.576 (0.350–0.948)	0.030	0.263 (0.154–0.449)	0.000	0.978 (0.498–1.917)	0.947
Surgical margin positive vs. negative	4.727 (2.188–10.214)	0.000	2.530 (1.056–6.059)	0.037	4.420 (1.364–14.318)	0.013	1.945 (0.532–7.227)	0.321

Abbreviation: SCC = squamous cell cancer; ADE = adenocarcinoma or adenosquamous carcinoma; DSI = depth of stromal invasion; HTB = high-grade tumor budding; LTB = low-grade tumor budding; LVSI = lymphovascular space invasion; PLNM = pelvic lymph node metastasis; PI = parametrium involvement; NA = not assessed; HR = hazard ratio; CI = confidence interval.

### Novel predictive models involving tumor budding for recurrence following radical surgery

**Fig 4:**
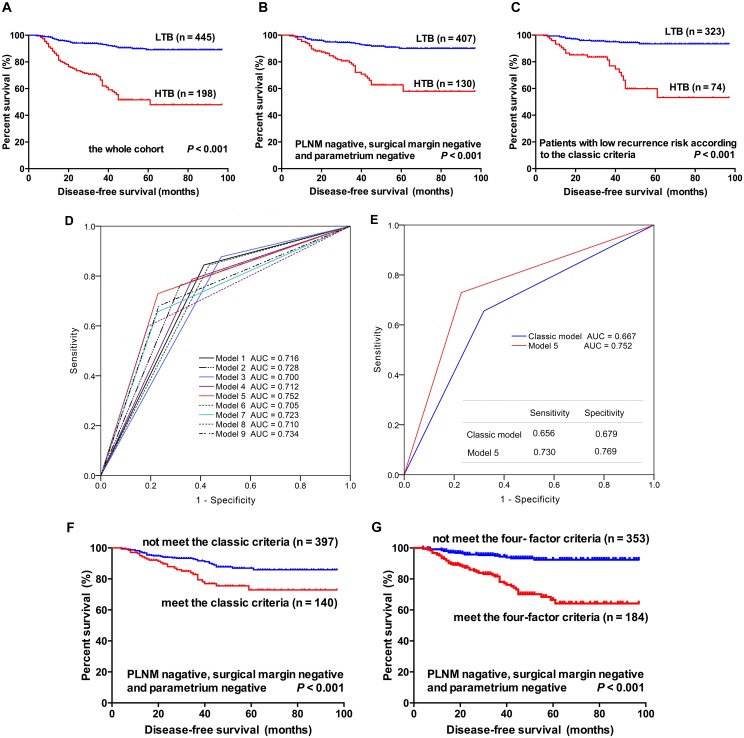
Novel predictive models involving tumor budding for recurrence following radical surgery. A-C, Kaplan-Meier curves for disease-free survival between ESCC patients with HTB and those with LTB. The presence of HTB had a significantly adverse effect on the disease-free survival in the whole cohort (A), patients without any of high-risk factors (B), and those with low recurrence risk according to the classic criteria (C). D-E, ROC curves for recurrence via multiple models. Of the nine models, Model 5 (HTB as an additional intermediate-risk factor with LVSI, DSI of outer 1/3, and tumor size ≥ 4 cm) exhibited the highest areas under the curve (AUC = 0.752; D); compared with the Classic model, Model 5 also had a larger AUC (0.752 versus 0.667; E). F-G, Kaplan-Meier curves for disease-free survival using different criteria. The four-factor criteria (G), using HTB as an additional intermediate-risk factor (at least two risk factors of HTB, tumor size ≥ 4 cm, DSI of outer 1/3, and LVSI), more effectively stratified the patients without any high-risk factor with recurrence risk than the Classic criteria (F).

Following the determination that tumor budding was an independent risk factor for recurrence in women with ESCC after radical surgery, the question arose as to whether the addition of budding to the classic risk factors improves the accuracy of recurrence prediction. The estimated 3-year rate was 66% in the HTB group versus 93% in the LTB group (log-rank test, *P* < 0.001; Fig 4A). We subsequently determined whether budding effectively stratified the patients with a recurrence risk in the patients without any high-risk factor (N = 537). In this subgroup of patients, Kaplan-Meier curves and log rank tests indicated that the disease-free survival in the HTB group was significantly lower than the LTB group (*P* < 0.001; Fig 4B). Moreover, of the 397 patients with a low risk for recurrence (according to the classic criteria), the HTB remained significantly associated with a worse disease-free survival than the LTB (3-year disease-free survival, 76% versus 95%, respectively, *P* < 0.001; Fig 4C). These findings suggest that HTB may improve the stratification strategy in women with a recurrence risk.

We subsequently established different criteria, including HTB and the classic risk factors, and compared their performances with the classic criteria using log-rank tests and ROC analysis. Nine models (termed Models 1–9) were established (Table 3): HTB as an additional high-risk factor to PLNM, parametrium involvement, and positive surgical margin with varied combinations of classic intermediate-risk factors (Models 1, 2, and 3); HTB as an additional intermediate-risk factor to tumor size (≥ 4 cm or ≥ 2 cm), DSI (outer 1/2 or outer 1/3), and LVSI (Models 4, 5, and 6); HTB as an obligate but not sufficient intermediate-risk factor, i.e., patients with HTB and at least one factor of large tumor size, DSI, and LVSI were considered at risk of recurrence (Models 7, 8, and 9). Of these models, Model 5, which included tumor size ≥ 4 cm, DSI of the outer 1/3, LVSI, and HTB as intermediate-risk factors, provided the highest performance as indicated by the log-rank tests and the ROC analysis (Table 3 and Fig 4D). Moreover, this four factor criteria had a larger AUC of 0.752 versus 0.667 in the classic model, accompanied with improved specificity and sensitivity (76.9% versus 67.9%, 73.0% versus 65.6%, respectively; Fig 4E). In the subgroup of patients without any high-risk factor, survival curves revealed that the four-factor criteria could more effectively stratify the patients at a substantial risk for recurrence compared with the classic three factor criteria (Fig 4F and 4G).

**Table 3:** Univariate analysis of various models predicting recurrence.

Models	High-risk factors*	Intermediate-risk factors*	ROC analyses		Log rank test	
			AUC	95% CI	Chi-square	*P* value
Classic	PLNM, PI, PSM	2CM, OUT 1/3, LVSI	0.667	0.611–0.723	50.679	0.000
HTB as an additional high-risk factor						
Model 1	PLNM, PI, PSM, HTB	2CM, OUT 1/3, LVSI	0.716	0.667–0.764	76.045	0.000
Model 2	PLNM, PI, PSM, HTB	4CM, OUT 1/3, LVSI	0.728	0.677–0.778	91.516	0.000
Model 3	PLNM, PI, PSM, HTB	2CM, OUT 1/2, LVSI	0.700	0.653–0.748	60.958	0.000
HTB as an additional intermediate-risk factor						
Model 4	PLNM, PI, PSM	2CM, OUT 1/3, LVSI, HTB	0.712	0.662–0.763	74.238	0.000
Model 5	PLNM, PI, PSM	4CM, OUT 1/3, LVSI, HTB	0.752	0.701–0.804	133.930	0.000
Model 6	PLNM, PI, PSM	2CM, OUT 1/2, LVSI, HTB	0.705	0.656–0.754	64.345	0.000
HTB as an obligate but not sufficient intermediate-risk factor**						
Model 7	PLNM, PI, PSM	2CM, OUT 1/3, LVSI, HTB	0.723	0.668–0.778	95.064	0.000
Model 8	PLNM, PI, PSM	4CM, OUT 1/3, LVSI, HTB	0.710	0.652–0.767	101.649	0.000
Model 9	PLNM, PI, PSM	2CM, OUT 1/2, LVSI, HTB	0.734	0.680–0.788	99.887	0.000

* Patients with one high-risk factor or two of intermediate-risk factors were regarded as with a risk for recurrence.

** Patients with HTB and at least one factor of large tumor size, DSI, and LVSI were considered at risk of recurrence.

Abbreviation: ROC = receiver operating characteristic curve; AUC = area under curve; CI = confidence intervals; PLNM = pelvic lymph node metastasis; PI = parametrium involvement; PSM = positive surgical margin; 2CM = tumor size ≥ 2 cm; OUT1/3 = stromal invasion of the outer third of the cervical wall; LVSI = lymphovascular space invasion; HTB = high-grading tumor budding; 4CM = tumor size ≥ 4 cm; OUT1/2 = stromal invasion of the outer half of the cervical wall.

## Discussion

In the ESCC cohort, tumor budding was strongly associated with poor survival following radical surgery, and HTB was identified as an independent prognostic risk factor for recurrence. More importantly, the integration of HTB with recognized risk factors significantly improved the risk stratification of the ESCC patients with an intermediate risk after primary radical surgery. The present study is the first research to identify the presence and prognostic value of tumor budding in ESCC. Moreover, we established a model that included tumor budding, tumor size, DSI, and LVSI to stratify ESCC patients at risk for recurrence who may benefit from an adjuvant therapy. These findings provide the first evidence that supports the implications of tumor budding in routine diagnostic pathology and the clinical management of ESCC.

In the present study, tumor budding was identified in most (91.3%) ESCC, and the number of buds was positively correlated with the FIGO stage, tumor size, DSI, LVSI, and PLNM. These findings suggest that tumor budding is a frequent early event of cervical cancer dissemination and is associated with cervical cancer progression. Similar results have been demonstrated in colorectal carcinoma [21, 26], breast cancer [16], and gastric cancer [27]. The budding cells at the invasive tumor front are considered epithelial mesenchymal transition (EMT)-derived tumor cells, with increased migratory and invasive capacities and stem cell properties [21, 28, 29]. During EMT, epithelial cells lose their cell polarity and cell-cell adhesion and gain a mesenchymal-like phenotype. EMT is recognized as a critic mechanism of tumor dissemination and metastasis in epithelial malignancies [30]. In cervical cancer, EMT plays a key role in PLNM; the onset of EMT increases the tumor stem cell subpopulation and leads to chemo-resistance and radio-resistance in cervical cancer [31]. In addition, the tumor budding-related immune microenvironment, e.g., FoxP3-positive lymphocyte infiltration, which comprises a subset of lymphocytes known to suppress the host immune response, may contribute to tumor invasiveness [14, 19]. In this context, the budding tumor cells represent a subpopulation with substantial potential to disseminate and metastasize at the invasive edge rather than “normal”, isolated tumor cells.

HTB has been demonstrated to independently affect disease-free survival and overall survival in colorectal cancers [12, 32], lung carcinomas [18, 19], and esophageal squamous cell carcinomas [13]. Moreover, HTB may serve as an additional prognostic factor to identify patients with stage I/II colorectal carcinoma at risk of recurrence following curative surgery [33, 34]. For the first time, we identified HTB as an independent risk factor for disease-free survival and overall survival in ESCC and provide evidence that supports its clinical use as an additional factor in the risk stratification of patients with ESCC following radical surgery. Despite the relatively large number of patients we studied, selection bias should not be ignored because of the single-center retrospective study design. Thus, the prediction model we proposed should be verified in an expanded cohort with longer follow-up; however, the contribution of HTB to improve the prediction of recurrence in ESCC patients following radical surgery is rather definite.

To date, adjuvant radiotherapy or concurrent chemo-radiation is the standard management for ESCC patients following radical surgery with a recurrence risk. However, tumor budding may lead to a poor response to therapy. EMT in cervical cancer has been associated with chemo-resistance and radio-resistance [31]. Tumor budding was predictive for non-response to anti-EGFR therapies in metastatic colorectal cancer patients [35]. In addition, tumor budding in rectal cancer biopsies prior to neoadjuvant therapy predicted a poor response to therapy [36]. Thus, prospective, randomized controlled trials are required to determine whether the use of HBT as an additional risk factor benefits ESCC patients undergoing surgery regarding disease-free survival and/or overall survival.

Tumor bud counting is technically feasible because it is based on H&E staining and Pan-cytokeratin immunostaining in cases of stromal inflammation or fibrosis at the invasive front [37]. In addition, the 2-tier division of the tumor bud count (HTB versus LTB) exhibited a high-level of inter-observer agreement [21], which facilitates the standardization of tumor bud scoring in ESCC and its implementation in routine diagnostic pathology. According to the ROC analysis results in the present study, we classified tumors of 5 buds/field or more as HTB. However, the cut-off value for the definition of HTB in ESCC may vary across different patient cohorts. In colorectal carcinoma, the cutoff values of tumor budding were ≥ 15 buds/field and ≥ 25 buds/field in two separate studies using ROC curve analysis [34, 35].

A precise stratification of patients is critical for tailoring the individualized management of disease. In the present study, we demonstrated that HTB was an independent adverse prognostic factor in ESCC following radical surgery; moreover, as an additional intermediate-risk factor, it significantly improves the stratification of patients with a recurrence risk in terms of both sensitivity and specificity. These findings support the impact of tumor budding on the clinical outcomes of ESCC and highlight its clinical relevance in guiding treatment decisions regarding adjuvant therapy in patients who underwent a radical hysterectomy and pelvic lymphadenectomy. Despite the promising prognostic value of tumor budding shown in our study, as we devote future research toward implementing tumor budding assessment into clinical trials of cervical cancer, we must standardize the scoring systems to improve accuracy and inter-observer agreement.

## Supporting Information

S1 File The data underlying the findings described in the study. (XLS)

S1 STARD Checklist STARD checklist for reporting of studies of diagnostic accuracy (*version 2015*). (DOCX)
